# An Evaluation of Psychogenic Predictors of Non-Organic Erectile Dysfunction

**DOI:** 10.3390/medicina59071195

**Published:** 2023-06-25

**Authors:** Osman Zulkif Topak, Zafer Sinik, Nalan Kalkan Oguzhanoglu, Tugce Toker Ugurlu

**Affiliations:** 1 Department of Psychiatry, School of Medicine, Pamukkale University, Denizli 20160, Turkey; 2 Department of Urology, Odak Hospital, Denizli 20100, Turkey

**Keywords:** erectile dysfunction, childhood trauma, attachment styles

## Abstract

*Background and Objectives:* Erectile dysfunction is a significant problem, which diminishes the quality of life. The aim of this study was to investigate the relationship of childhood trauma and attachment styles in the aetiology of psychogenic erectile dysfunction. *Materials and Methods:* The study included 80 participants (40 patients who presented with the complaint of erectile dysfunction, were not determined with an organic pathology, and were diagnosed with erectile dysfunction according to the DSM-5 criteria; and a control group of 40 healthy subjects.) The structured clinical interview form for DSM-5 (SCID-5) was applied to all the participants, together with the International Erectile Function Index (IIEF), the Childhood Trauma Questionnaire (CTQ), the Relationship Scale Questionnaire (RSQ), and the Beck Depression Inventory (BDI). *Results:* The emotional abuse (*p* = 0.002), physical abuse (*p* = 0.049), emotional neglect (*p* = 0.004), physical neglect (*p* = 0.002), and total scale points of the CTQ were determined to be significantly higher in the patient group than in the control group. Secure (*p* = 0.022) and dismissive (*p* = 0.009) attachment styles were found to be higher in the control group. As the time together with the current sexual partner increased, so the severity of erectile dysfunction increased, and sexual function, orgasmic function, sexual satisfaction, and general satisfaction decreased. As emotional abuse, sexual abuse, and physical neglect increased, the severity of erectile dysfunction increased. Childhood trauma (*β* = −0.275, *t* (73) = −2.704, *p* = 0.009) and the duration together with the partner (*β* = −0.249, *t* (73) = −2.512, *p* = 0.014) were found to be predictive of erectile dysfunction. *Conclusions:* The results of this study demonstrated that childhood trauma and the time elapsed without treatment are predictors of psychogenic erectile dysfunction severity, and secure attachment style and self-esteem play an important role in the aetiology of psychogenic erectile dysfunction.

## 1. Introduction

Sexual health has become more important in recent years, as a deterioration in sexual health could be an indicator of future general health problems [1]. Sexual dysfunction is not only a problem in achieving pleasure or satisfaction, but it is also a source of many psychosocial problems [2]. It creates feelings in people of being unloved, disapproval of femininity or masculinity, loss of confidence, embarrassment, and lack of pride [3].

Erectile dysfunction is the most frequently encountered sexual dysfunction in males and can be defined as an insufficient duration and severity of penile stiffness at the stage of sexual stimulation [4]. As a problem which is very frequently encountered, with a global prevalence of 52% [5], erectile dysfunction was previously accepted as a problem of old age, but studies in recent years have reported that it is often seen in males aged <40 years [6,7]. Although a common problem in society, the rates of presentation for a solution are low [4]. Erectile dysfunction is a significant problem, which diminishes the general quality of life of the individual and impairs self-esteem and close relationships with a partner [8].

There is generally not one single factor in the aetiology of erectile dysfunction, but a complex relationship of organic, psychogenic, or sociocultural reasons. It is known that normal penile erection is a neurovascular phenomenon coordinated by endocrine, vascular, and nervous system and controlled by emotional or psychological factors [9]. Although the causes of organic-origin erectile dysfunction can be determined more easily, the formation of psychogenic erectile dysfunction is not fully understood. Therefore, we need to know more about the psychological factors. Childhood sexual trauma, problems of sexual identity, unresolved partner or parental attachment styles, and religious or cultural taboos have been suggested as reasons for the early development of psychogenic erectile dysfunction [10].

Childhood trauma is a situation, which harms the physical and mental health of the child and has repercussions in adult life. Mass problems, such as wars, natural disasters, and diseases, in the twentieth century had a destructive effect on children’s lives and found a wide field of study. Traumas can occur not only with mass experiences but also with human abuse or neglect. Child abuse and neglect are all the actions and inactions, which are inappropriate or harmful to the child and disrupt or limit the biopsychosocial development. Childhood trauma is a societal problem, which can have tragic consequences for the psychological development of an individual and severely destructive biopsychosocial outcomes, and it affects many areas of the public [11,12,13]. Following abuse and neglect, these children are at a high risk of developing psychiatric disorders in later years, especially mood and anxiety disorders [12,14]. Moreover, they are also at a high risk of sexual dysfunction. For example, histories of childhood sexual trauma were found to be associated with ejaculatory dysfunction [15]. At the same time, by affecting the relationship of the child with an object of attachment, childhood trauma can cause a basic loss of trust, resulting in attachment problems, and it can lead to significant psychopathologies [16]. According to the attachment theory, the attachment style established in infancy is reflected in the romantic relationships established in adulthood [17]. Insecure attachment styles formed for any reason in childhood can lead to several psychopathologies. The psychological disorders that develop could be related to sexual function problems, such as psychogenic erectile dysfunction in males [10]. The use of drugs that use similar pathways can also be a proof of this. So far, one of the drugs targeting the central nervous system, which have been used for the erectile dysfunction treatment in men, is serotonin reuptake inhibitor trazodone. Additionally, the agonists of melatonin, which is synthesised by serotonin, were found to be capable of inducing repeated penile erection episodes in human male volunteers and in men with psychogenic impotence [18]. Childhood trauma experienced in the family environment and the attachment style established with parents may be a factor in the emergence of non-organic-origin erectile dysfunction. If it is considered that there is a relationship between parental attachment style and sexual function disorders, it can be said that not many empirical studies have been conducted on this subject [19].

In the light of all this information, it was predicted that childhood trauma and attachment styles could play a role in the early development of psychogenic erectile dysfunction. The hypotheses of this study were that childhood traumas are more common in individuals with psychogenic erectile dysfunction than in healthy controls, and psychogenic erectile dysfunction is associated with attachment styles.

The aim of this study was to investigate the relationship between psychogenic erectile dysfunction which emerged in adulthood and childhood trauma and attachment styles. The study results will be able to help in the determination of the aetiology of this important public health problem and could facilitate treatment of the problem.

## 2. Materials and Methods

### 2.1. Study Participants and Design

The study included males aged 18–65 years who presented at the Urology Polyclinic between January 2023 and April 2023 with complaint of an erectile disorder from the beginning of the relationship (at least 6 months), were not determined with an organic pathology according to laboratory test results and a physical examination carried out by a urology specialist, and were diagnosed with erectile dysfunction according to the DSM-5 criteria in a structured psychiatric interview conducted by a psychiatry specialist. Every patient had physical examination focused on genitourinary (penile deformities, prostatic diseases, signs of hypogonadism), cardiovascular, endocrine, and neurological status to rule out organic erectile dysfunction. Physical examination also included the measurement of blood pressure and body mass index to assess abdominal obesity. Laboratory evaluation was conducted based on the fasting glucose level for diabetes, a lipid panel for hyperlipidaemia, and a morning sample of total testosterone. Additional laboratory tests were carried out, such as thyroid-stimulating hormone, gonadotropin, and prolactin levels. Medical and surgical history, previous consultations and treatments, use of medications and other substances were also evaluated to exclude organic aetiology. Patients with no medical comorbidity and risk factor for organic erectile dysfunction and with normal nocturnal erections constituted the study population [20]. The patients included had no other physical or psychological disease, were not using any drugs that could affect sexual function, were in a relationship with a regular partner, and consented to voluntary participation in the study. The control group was formed of healthy males in the 18–65 years age range, age-matched to the patient group, with no erectile dysfunction, no physical or psychological disease, in a relationship with a regular partner, and with a score of ≥26 on the Turkish version of the International Erectile Function Index (IIEF). The calculated power (1-beta) was 0.8, considering type 1 error (alfa) of 0.05, sample size of 40, and effect size of 0.55.

Sociodemographic information, such as age, marital status, and education level, was collected for all participants on a Sociodemographic Data Form. The structured clinical interview form for DSM-5 (SCID-5) was administered, together with the IIEF Turkish version, the Childhood Trauma Questionnaire (CTQ), the Relationship Scales Questionnaire (RSQ), and the Beck Depression Inventory (BDI).

### 2.2. Measurement Tools

International Erectile Function Index (IIEF): The IEFF is one of the scales frequently used to evaluate erectile function, sexual function, orgasmic function, sexual satisfaction, and general satisfaction in males [21]. The validity of the scale in Turkish has been shown in Turkish Andrology Society studies [22,23]. From a total of 30 points in the IIEF, a score of 0–10 points is evaluated as severe erectile dysfunction, 11–16 points as moderate, 17–21 points as mild–moderate, 22–25 points as mild, and 26–30 points as normal.

Childhood Trauma Questionnaire (CTQ): The CTQ is a measurement tool with proven reliability and validity, which has been shown to be useful in the retrospective and quantifiable evaluations of experiences related to abuse and neglect before the age of 20 years [24]. The validity and reliability studies of the scale in Turkish were conducted by Sar et al. [25]. Evaluations are made in the subscales of emotional abuse, physical abuse, physical neglect, emotional neglect, and sexual abuse. The recommended cut-off points are 5 points for sexual and physical abuse, 7 points for physical neglect and emotional abuse, 12 points for emotional neglect, and 35 points for the total score.

Relationship Scales Questionnaire (RSQ): The RSQ was developed by Griffin and Bartholemew [26] to measure the four attachment styles of secure, dismissive, fearful, and preoccupied. The validity and reliability studies of the scale in Turkish were conducted [27]. In the 30-item RSQ, the subject marks on a 7-point scale the degree to which they identify their general attitude to themselves and to close relationships. According to the highest attachment style points, the subjects are grouped as secure, dismissive, fearful, or preoccupied attachment style.

Beck Depression Inventory (BDI): The BDI was developed to measure the degree of somatic, emotional, cognitive, and motivational symptoms of depression in adolescents and adults, and to monitor changes with treatment [28]. This self-reported Likert-type scale consists of 21 items. The scores are interpreted as 0–9 points: minimal, 10–16: mild, 17–29: moderate, and 30–63: severe. The validity and reliability studies of the scale in Turkish were conducted [29].

### 2.3. Statistical Analysis

The data obtained in the study were analysed statistically using SPSS vn. 22.0 software (Statistical Package for Social Sciences). The conformity of the data to normal distribution was assessed with the Kolmogorov–Smirnov test and the skewness–kurtosis coefficients. Descriptive statistics were stated as mean ± standard deviation (SD) values for continuous variables and as number (*n*) and percentage (%) for categorical data. For the evaluation of differences between independent groups of quantifiable data, the Student’s *t*-test was used when parametric conditions were met, and the Mann–Whitney U-test was used when they were not met. In the comparisons of categorical data, the Pearson chi-square test and Fisher’s exact test were applied. The relationships between variables were examined with Pearson correlation analysis when data showed normal distribution and with Spearman correlation analysis when data distribution was not parametric. Multiple logistic regression analysis was applied in the evaluation of risk for categorical independent variables. All the results were given in a 95% confidence interval, and a value of *p* < 0.05 was accepted as the level of statistical significance.

### 2.4. Ethical Approval

All the study participants provided informed consent for participation in the study in accordance with the Helsinki Declaration. The approval for the study was granted by the Non-Interventional Clinical Research Ethics Committee of the University (decision no: 19, dated: 27 December 2022).

## 3. Results

### 3.1. Sociodemographic Data

The distribution of the sociodemographic data of the patient and control groups is shown in Table 1. The total sample of 80 males had a mean age of 39.28 ± 7.43 years (range, 29–62 years), comprising 40 (50%) males in the patient group with a mean age of 40.85 ± 7.28 years (range, 29–55 years) and 40 in the control group with a mean age of 37.72 ± 7.34 years (range, 29–62 years). The mean age was similar in the two groups (*p* = 0.06). In the patient group, 35% (*n:* 14) had an education level of primary school, and in the control group, 62.5% (*n:* 25) were university graduates (*p* = 0.004). The place of residence was reported as a rural area by 22.5% (*n:* 9) of the patient group and by 2.5% (*n:* 1) of the control group, and the difference was statistically significant (*p* = 0.007). The marital status of the patient and control groups was determined to be similar, as 92% (*n:* 37) and 90.0% (*n*: 36), respectively, were married. The time with the current sexual partner was determined to be statistically significantly longer in the patient group (167.40 ± 100.30 months (range, 12–384 months)) than in the control group (112.57 ± 92.33 months (range, 12–506 months)) (*p* = 0.013).

### 3.2. Evaluation of the Scales

From the evaluation of the IIEF, erectile dysfunction was determined in the patient group at a mild–moderate level in 15 (37.5%) patients, a moderate level in 13 (32.5%), a mild level in 8 (20%), and a severe level in 4 (10%). The presence of childhood trauma was determined at a significantly higher rate in the patient group (*n:* 23, 57.5%) than in the control group (*n:* 13, 32.5%) (*p* = 0.025). In the evaluation of the attachment styles, the dismissive style was determined at the highest rate in the patient group (*n:* 18, 45%), and the secure style was determined at the highest rate in the control group (*n:* 19, 47.5%) (*p* = 0.003).

**Figure 1:**
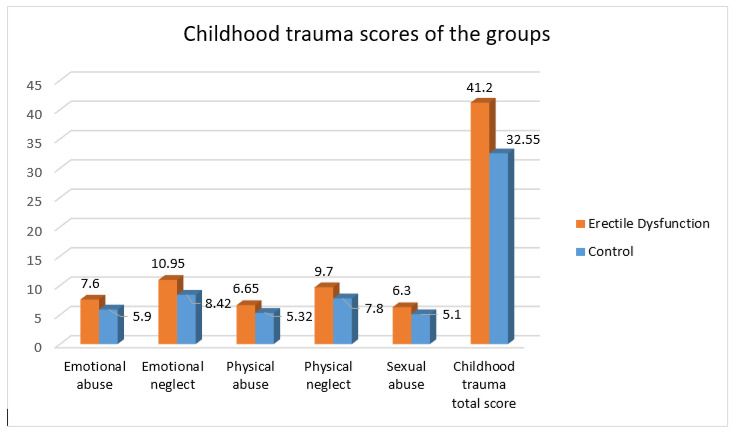
Childhood trauma scores of the groups.

The comparisons of the scale data between the two groups are shown in Table 2. The orgasmic function, sexual function, sexual satisfaction, and general satisfaction points were determined to be statistically significantly higher in the control group than in the patient group (*p* < 0.001). The childhood trauma scale total points (*p* = 0.001), emotional abuse (*p* = 0.002), physical abuse (*p* = 0.049), emotional neglect (*p* = 0.004), and physical neglect (*p* = 0.002) points were determined to be significantly higher in the patient group than in the control group (Figure 1). The rate of sexual abuse was seen to be similar in both groups (*p* = 0.066). In the comparisons made according to the attachment style, no significant difference was determined between the groups with respect to fearful (*p* = 0.117) and preoccupied (*p* = 0.131) attachment, and a significantly higher rate of secure (*p* = 0.022) and dismissive (*p* = 0.009) attachment styles was determined in the control group than in the patient group.

Correlations between the erectile dysfunction points and the scale points were examined in all the study participants. The erectile dysfunction points were determined to be negatively correlated with the time with the current sexual partner at a moderate level (r = −0.283, *p* = 0.011) and positively correlated with orgasmic function points at a very strong level (r = 0.849, *p* < 0.001), with sexual function points at a strong level (r = 0.749, *p* < 0.001), with sexual satisfaction at a very strong level (r = 0.877, *p* < 0.001), and with general satisfaction at a very strong level (r = 0.844, *p* < 0.001). As the time together with a sexual partner increased, so the severity of erectile dysfunction increased, and sexual function, orgasmic function, sexual satisfaction, and general satisfaction decreased. When the correlations with the CTQ and subscale points were examined, negative correlations were determined between the erectile dysfunction points and emotional abuse at a moderate level (r = −0.331, *p* = 0.003), sexual abuse at a weak level (r = −0.224, *p* = 0.046), physical neglect at a moderate level (r = −0.329, *p* = 0.003), and the total CTQ points at a moderate level (r = −0.315, *p* = 0.004). Accordingly, it was determined that as the emotional abuse, sexual abuse, and physical neglect increased, so the severity of erectile dysfunction increased. Further analysis could not determine which type of childhood trauma has the greatest impact on adult erectile function. From the attachment style scale points, positive correlations were determined between erectile dysfunction and fearful attachment at a weak level (r = 0.245, *p* = 0.028) and dismissive attachment at a moderate level (r = 0.260, *p* = 0.020). All the correlations are shown in Table 3.

### 3.3. Multiple Linear Regression Model

The results of the multiple linear regression analysis performed to examine the effect of independent variables on erectile dysfunction are shown in Table 4. The time together with the current sexual partner, the CTQ total points, and the secure, dismissive, fearful, and preoccupied attachment styles scale points were analysed as independent variables, and the erectile dysfunction scale points as the dependent variable. In the significant regression model (*F*(6, 73) = 4.940, *p* < 0.001) formed as a result of the analysis, 23% of the variance in the dependent variable was explained by independent variables (R^2^_adjusted_ = 0.230). This demonstrated that erectile dysfunction was affected by the time together with the current sexual partner and by the CTQ total scale points. The CTQ total scale points significantly and negatively predicted erectile dysfunction (*β* = −0.275, *t* (73) = −2.704, *p* = 0.009). The time together with the current sexual partner significantly and negatively predicted erectile dysfunction (*β* = −0.249, *t* (73) = −2.512, *p* = 0.014).

This section may be divided into subheadings. It should provide a concise and precise description of the experimental results, their interpretation, as well as the experimental conclusions that can be drawn.

## 4. Discussion

This study can be considered of importance with respect to demonstrating the relationship of psychogenic erectile dysfunction with childhood trauma and attachment style and demonstrating that childhood trauma and the time together with a partner can negatively predict erectile dysfunction, independently of all other variables.

In the comparison of the demographic data of the patient and control groups in this study, it was found that the patient group had a lower education level and lived in a rural area more frequently. Although not included in the study data, it can be assumed that cultural help may be sought more until presentation to a doctor, or that with a traditional perspective and accepting approach, there may be a delay in being able to express the problem. That more of the control group lived in the city and the education level was high could be explained by the greater participation of less conservative individuals, as participation in research related to sexuality would be more socially acceptable to this group. Akkuş et al. [22] reported that sociodemographic factors, such as a low level of education or income and living alone, could increase erectile dysfunction. Insufficient sexual knowledge associated with a traditional and conservative upbringing in rural areas, improper sexual behaviours, being compelled by exaggerated sexual expectations, and feelings of shame and guilt can cause the development of psychogenic erectile dysfunction [4].

In contrast to the effect on females, childhood trauma as a risk factor in the development of sexual function disorders is not clearly known [30]. Xu et al. suggested that the interrelation between autonomic nervous system and hypothalamic–pituitary–adrenal axis activity might enhance the comprehension of how stress affected the physical and mental health of psychogenic erectile dysfunction patients [31]. It has also been reported that sexual abuse can lead to problems related to erection, ejaculation, and orgasm in males and performance anxiety [32]. Pulverman et al. [30] reported that the relationship between sexual function and sexual abuse was affected by many factors, such as sexually mediated cognitions, family dynamics, the level of daily stress, sympathetic activation, body image, and feelings of guilt and shame. In a study by Najman et al. [33], a correlation was determined between sexual function disorders and sexual abuse, and this relationship was shown to be more significant in females than in males. Gewitz-Meydan et al. pointed to the potential contribution of traumatic sexuality symptoms to sexual difficulties among survivors of child sexual abuse and lent support to the idea of offering trauma-focused therapy when treating the sexual difficulties of child sexual abuse survivors [34]. In contrast, in a study of university students by Kinzl et al. [35], it was shown that emotional neglect and physical abuse increased the risk of sexual dysfunction, but despite the correlation of core family relationships with the disorder, no correlation was determined with sexual abuse. One of the most significant findings of the current study was that the physical abuse, emotional abuse, physical neglect, emotional neglect, and total trauma points were found to be significantly higher in the group with psychogenic erectile dysfunction than in the group without erectile dysfunction. Although no difference was found in the sexual abuse points compared to the control group, sexual abuse was determined to be correlated with erectile dysfunction severity in the correlation analysis. These findings support the view that the type of childhood trauma could potentially have a place in the aetiopathogenesis of psychogenic erectile dysfunction. Therefore, sexual abuse in childhood can result in the two extremes of avoiding adult sexuality or excessive sexuality [36]. The reason for this may be that in addition to sexual abuse, several different factors, such as having experienced physical or emotional abuse, or abuse from an attachment figure or from outside the family, a broken parental relationship, or a problematic relationship with the spouse, can lead to varying outcomes in adulthood [37]. Another view is that when problems are experienced with sexuality, the individual may try to tolerate this by enacting a different attachment style [38]. There may be a subconscious effort to desensitise to reduce the emotional stress of re-living the traumatic event.

It has been reported in the literature that by leading to attachment problems, the trauma experienced in childhood can result in sexual dysfunction [32,39]. According to the attachment theory, interactions in early life affect the beliefs and expectations in adult relationships and play an important role in determining the emotional and sexual behaviour with the spouse [19,40,41]. After it was suggested that attachment styles could affect romantic relationships, it was thought that impaired attachment styles could be important in the aetiology of psychogenic erectile dysfunction by causing neurochemical and neuroendocrine changes or by leading to depression and anxiety [42]. Brassard et al. [43] determined higher rates of erectile dysfunction in males with an insecure attachment style compared to those with secure attachments. Individuals with low levels of anxiety and avoidance in adult attachment are evaluated as having a secure attachment style in close relationships [19]. The feeling of security created when attachment figures are accessible and can continuously and consistently respond to the demands of an infant will enable secure bonds to be established with others in the future. If this feeling is not created, insecure attachment will form through the development of secondary attachment strategies [40]. After insecure attachment develops, the anxiety increases, and increased anxiety reduces sexual function [44], causing decreased stimulation and orgasmic response in males and females [32,45]. It has been stated that insecure attachment types lead to less satisfaction in sexual relationships, more sexual dysfunction, and frequent different sexual relationships and sexual motivations [19,32].

Consistent with the literature, the rate of secure attachment was found to be significantly high in the control group in the current study. Although the high rate of dismissive attachment in the current study control group can be seen as an interesting finding, the common point of both these attachment types is the characteristic of high self-esteem. Therefore, the importance of self-esteem in erectile dysfunction is emphasised by the results of this study. Individuals with a secure attachment style have a positive perception of both themselves and others. As they feel worthy of love, think others are to be trusted, and have positive expectations of support and good intentions, they are able to establish closeness with others and remain autonomous. Individuals with an insecure attachment style have negative perceptions of others despite a positive self-perception. Their self-esteem is high, but as they fear being hurt, they do not want to trust others or become emotionally attached. In the results of the current study, the fact that dismissive attachment was found to be low in addition to secure attachment among patients with psychogenic erectile dysfunction showed that these individuals had low self-esteem. Psychogenic erectile dysfunction may develop in these individuals because of performance anxiety stemming from low self-esteem. Ciocca et al. [46] also reported that self-esteem was low in men with psychogenic erectile dysfunction.

In a study by Dunkley et al. [47], a correlation was found between fearful attachment and all aspects of sexual function. Rajkumar [42] also reported in a study of males with psychogenic erectile dysfunction that patients who may have an attachment problem were more anxious. These individuals who have an attachment with a high level of anxiety may continue relationships despite not being satisfied because of the fear of separation and abandonment [19]. In the correlation analyses of the current study, it was observed that as the time together with the current partner increased, so there was a decrease in sexual function, orgasmic function, sexual satisfaction, and general satisfaction, and that the time together with the sexual partner was a predictor of erectile dysfunction. Moreover, as the duration of the relationship increases, the severity of erectile dysfunction may be associated with decreased excitement in long-term relationships, the normalisation of the relationship, or the use of passive sexual life to improve the quality of the relationship.

There were some limitations to this study—primarily the low number of cases—and the fact that the data were based on self-reports means that the results cannot be generalised. Further multicentre studies with greater numbers of patients would be able to better clarify the relationship of childhood trauma and attachment styles with psychogenic erectile dysfunction. The cross-sectional design of the study was another limitation of this study. There is a need to know more about the treatment process of these patients. Follow-up studies can contribute more to the literature on this issue. Nevertheless, the results of this study can be considered of value, as the relationship of erectile dysfunction with childhood trauma and attachment styles was compared with a control group, and there have been few studies of this subject in the literature.

## 5. Conclusions

In conclusion, the results of this study demonstrated that childhood trauma and time elapsed without treatment were predictive in the aetiology of psychogenic erectile dysfunction, independently of all other variables, as well as supporting the hypothesis that a secure attachment style and self-esteem play an important role in psychogenic erectile dysfunction. For patients presenting with a psychogenic erectile dysfunction, the detailed questioning of a history of childhood trauma and attachment style could help in the planning of more detailed and different therapy approaches to treatment in order to be able to reach a better outcome.

## Figures and Tables

**Table 1:** Sociodemographic and clinical characteristics of the groups.

		Patient	Control	*p*
		*n* (%)	*n* (%)	
Education	Primary	14 (5)	3 (7.5)	0.004 *
	High School	13 (32.5)	12 (30.0)	
	University	13(32.5)	25 (62.5)	
Living	Urban	31 (77.5)	39 (97.5	0.007 *
	Rural	9 (22.5)	1 (2.5)	
Marital Status	Married	37 (92.5)	36 (90)	1.000 **
	Single	3 (7.5)	4 (10)	
Living With Partner	Together	40 (100)	39 (97.5)	1.000 **
	Apart	0 (0)	1 2.5	
		**Mean ± SD**	**Mean ± SD**	
Age		40.85 ± 7.28	37.72 ± 7.34	0.06 ***
Duration of Relation With Current Sexual Partner (Months)		167.40 ± 100.30	112.57 ± 92.33	0.013 ***

* Pearson’s Chi Square test, ** Fisher’s Exact test, *** T-test.

**Table 2:** Comparison of the scale and subscale scores of the groups.

Scales	Patient	Control	*p*
Erectile function index total score	17.02 ± 5.25	28.57 ± 1.43	<0.001 *
Orgasmic function	6.02 ± 2.40	9.50 ± 0.81	<0.001 *
Sexual function	5.72 ± 1.82	7.87 ± 1.01	<0.001 *
Sexual satisfaction	6.85 ± 2.71	11.82 ± 1.79	<0.001 *
General satisfaction	5.57 ± 1.93	8.80 ± 1.26	<0.001 *
Childhood trauma scale total score	41.20 ± 13.86	32.55 ± 5.51	0.001 **
Emotional abuse	7.60 ± 2.88	5.90 ± 1.49	0.002 *
Emotional neglect	10.95 ± 4.44	8.42 ± 2.89	0.004 *
Physical abuse	6.65 ± 3.08	5.32 ± 0.79	0.049 **
Physical neglect	9.70 ± 2.94	7.80 ± 2.18	0.002 *
Sexual abuse	6.30 ± 3.48	5.10 ± 0.44	0.066 **
Secure attachment	4.12 ± 0.91	4.59 ± 0.90	0.022 *
Fearful attachment	3.63 ± 1.09	4.00 ± 0.98	0.117 **
Preoccupied attachment	3.81 ± 0.80	3.50 ± 1.04	0.131 *
Dismissive attachment	4.21 ± 1.17	4.81 ± 1.10	0.022 *

* T-test, ** Mann–Whitney U-test.

**Table 3:** Correlations between erectile function index total score and age, duration of relation, and other scales.

		Erectile Function Index Total Score
Age	r	−0.192
	p	0.088
Duration of relation with current sexual partner (months)	r	−0.283
	p	0.011
Orgasmic function	r	0.849
	p	<0.001
Sexual function	r	0.749
	p	<0.001
Sexual satisfaction	r	0.877
	p	<0.001
General satisfaction	r	0.844
	p	<0.001
Childhood trauma scale total score	r	−0.315
	p *	0.004
Emotional abuse	r	−0.331
	p	0.003
Emotional neglect	r	−0.212
	p	0.060
Physical abuse	r	−0.203
	p *	0.071
Physical neglect	r	−0.329
	p	0.003
Sexual abuse	r	−0.224
	p *	0.046
Secure attachment	r	0.121
	p	0.258
Fearful attachment	r	0.245
	p	0.028
Preoccupied attachment	r	−0.138
	p	0.222
Dismissive attachment	r	0.260
	p	0.020

p = Pearson correlation test, * Spearman correlation test.

**Table 4:** Multiple linear regression analysis results of predictors of erectile dysfunction.

Scales	Erectile Function Index				
	B	SH	β	%95 CI	*p*
Duration of relation with current sexual partner (months)	−0.017	0.007	−0.249	−0.031–−0.004	0.014
Childhood trauma scale total score	−0.168	0.062	−0.275	−0.292–−0.044	0.009
Secure attachment	1.408	0.801	0.189	−0.189–3.004	0.083
Fearful attachment	1.222	0.812	0.184	−0.396–2.840	0.137
Preoccupied attachment	−0.714	0.754	−0.097	−2.217–0.790	0.347
Dismissive attachment	0.962	0.712	0.163	−0.456–2.380	0.181

*F* (6–73) = 4.940, *p* < 0.001—(R^2^_adjusted_ = 0.230).

## Data Availability

The data sets used and/or analysed during the current study are available from the corresponding author upon reasonable request.
